# Choroidal metastases from thoracic cancer: a retrospective study on clinical characteristics and treatment efficacy

**DOI:** 10.3389/fmed.2025.1491278

**Published:** 2025-02-26

**Authors:** Xiacheng Lin, Yusheng Zhong, Haiping Li, Yating Yang, Ahui Liu, Yajing Shi, Jianhong Liang, Yong Cheng

**Affiliations:** ^1^Center of Optometry, Department of Ophthalmology, Peking University People's Hospital, Institute of Ophthalmic and Optometric Medicine, Beijing Key Laboratory of Diagnosis and Therapy of Retinal and Choroid Diseases, Optometry School of Peking University Health Science Center, Beijing, China; ^2^Beijing Key Laboratory of Restoration of Damaged Ocular Nerve, Peking University Third Hospital, Beijing, China

**Keywords:** choroidal metastases, ocular tumors, tumor metastasis, thoracic cancer, bevacizumab, plaque radiotherapy, transpupillary thermotherapy

## Abstract

**Objective:**

This study aimed to examine the clinical characteristics of choroidal metastases (CMs) and assess the efficacy of various treatment strategies for CMs in a specific patient cohort.

**Methods:**

This retrospective case series study included 32 patients (38 eyes) diagnosed with CM at the Department of Ophthalmology, Peking University People’s Hospital, between 2009 and 2022. The cohort included 10 male patients (10 eyes) and 22 female patients (28 eyes), with a mean age of 52.53 ± 10.81 years. Detailed medical histories and multiple ophthalmic examinations were performed for all patients, with diagnoses confirmed by two senior ophthalmologists. Clinical characteristics, treatment responses, and follow-up outcomes were analyzed.

**Results:**

Lung cancer was the most common primary tumor (53.1%), with six patients initially presenting with ocular symptoms. Initial ultrasound imaging revealed a mean tumor height of 3.02 mm and a mean basal diameter of 11.09 mm. Optical coherence tomography (OCT) revealed irregular, highly reflective foci with undulating anterior surfaces, and fluorescein angiography (FFA) revealed early choroidal tumor masking and late-phase mottled hyperfluorescence with occasional leakage. Indocyanine green angiography (ICGA) revealed hypofluorescence in early and late phases, with slightly mottled hyperfluorescence in the late phase. The follow-up period ranged from 3 months to 4 years (median 4 months), during which systemic and local treatments effectively controlled or delayed tumor progression in most patients.

**Conclusion:**

Lung and breast cancers are the leading sources of CMs, with bilateral or multifocal lesions more frequently linked to breast cancer. Treatment for CMs should be meticulously individualized and should take into account the patient’s overall condition, tumor burden, and precise tumor location. Local treatment is essential for patients with significant ocular symptoms. A combination of local and systemic treatments has been shown to lead to a more significant reduction in tumor burden.

## 1 Introduction

Choroidal metastases (CMs) are characterized by the spread of malignant tumor cells from distant sites to intraocular tissues via hematogenous or lymphatic routes. The choroid represents the most common site for intraocular metastasis, accounting for 88–89% of uveal metastasis cases, thereby far surpassing the involvement of the iris and ciliary body (1, 2). The primary malignancies have been found to originate from the breast (47%), lung (21%), digestive system (4%), kidney (2%), skin (2%), prostate (2%), or other regions (4%) (3).

Advancements in cancer treatment have extended patient survival, thus leading to an increase in the detection rates of CM (4). Current treatment approaches for CM encompass both systemic and local therapies. Among patients with extensive metastatic disease, ocular treatments are considered palliative measures aimed at preserving vision and alleviating pain progression, thus ultimately enhancing the quality of life (5). Shah, S. U. et al. reported that 44% of lung cancer patients with uveal metastasis were not diagnosed with lung cancer at their initial ophthalmic consultation (6). Given that the fundus findings of CM can mimic other ocular disorders (7), it is necessary to further clarify the clinical characteristics of CM to enhance early detection and increase the accuracy of diagnosing the primary malignancy.

A retrospective analysis was conducted on 32 patients with CMs who were treated at Peking University People’s Hospital. The clinical features were summarized, and the efficacy of various treatment modalities was systematically evaluated. This study aimed to provide valuable insights regarding the early detection, diagnosis, and clinical management of CM.

## 2 Materials and methods

### 2.1 Patients

**Figure 1:**
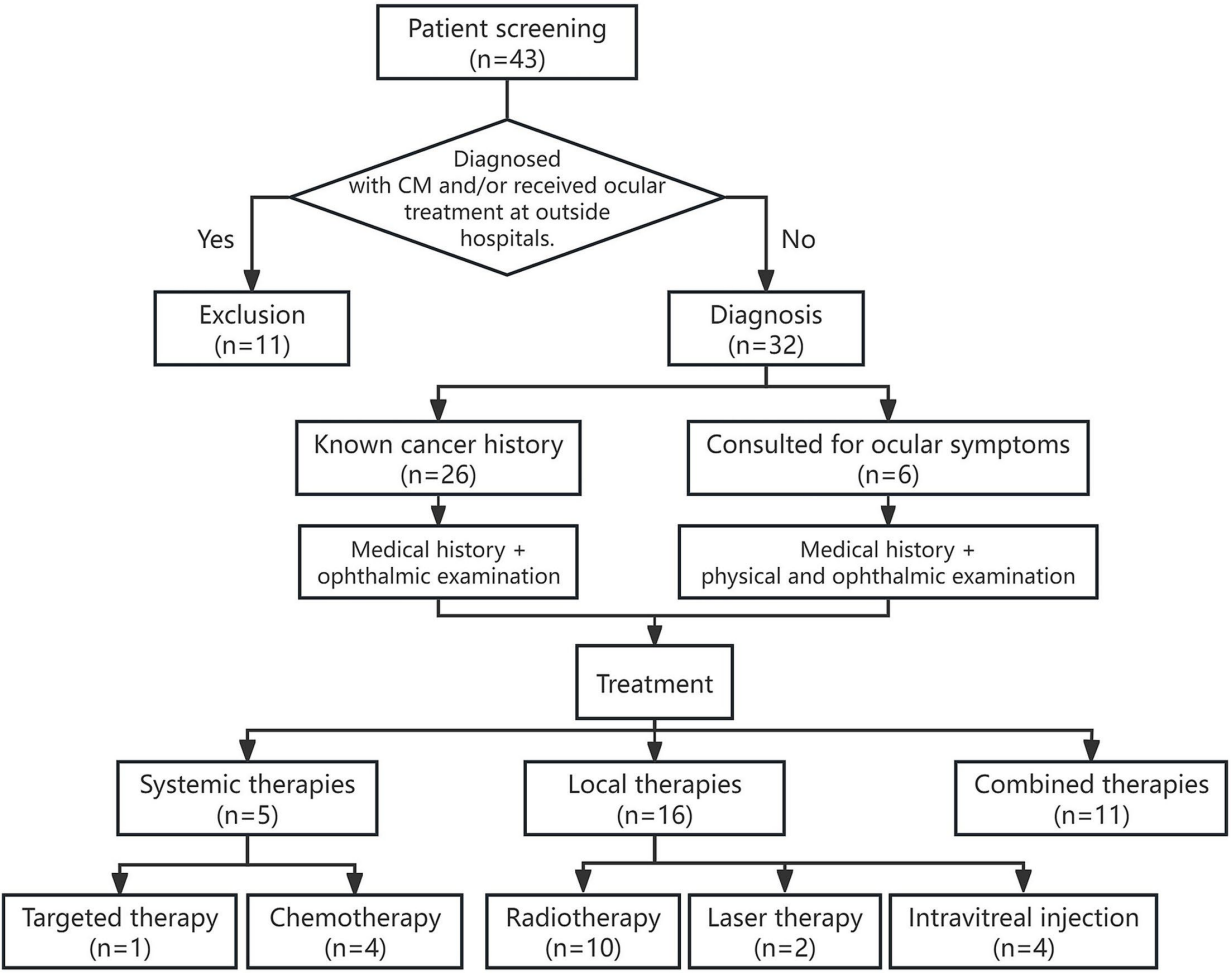
Flowchart of patient selection, diagnosis, and treatment process in a retrospective study of CM.

This retrospective case series was approved by the Ethics Committee of Peking University People’s Hospital (Ethics No.: 2015PHA037). A total of 32 patients (38 eyes) who were diagnosed with and treated for CM between November 2009 and July 2022 were included. The inclusion criterion required a diagnosis of CM by two senior ophthalmologists and confirmed by clinical follow-up lasting at least 3 months. Patients who were already diagnosed with CM and received ocular treatment at outside hospitals were excluded. The diagnostic criteria for CM were based on the patient’s cancer history and the presence of systemic metastasis at the time of CM diagnosis. Specifically: (1) In cases with a known history of metastatic disease, the past medical history combined with ophthalmic examination was typically sufficient for diagnosis. (2) In patients with a cancer history but no prior metastasis, diagnostic tests specific to the suspected cancer type were performed in addition to ophthalmic examination. (3) In the absence of a known primary tumor, a thorough medical history and physical examination were conducted to guide the ophthalmologist in identifying the primary tumor. Biopsy was considered when it was necessary for cytological or histopathological analysis (3) (see Figure 1 for details).

Informed consent was obtained from all patients prior to enrollment. The following clinical data were collected: laterality (left, right, or bilateral), visual acuity (VA), intraocular pressure, slit-lamp microscopy, indirect ophthalmoscopy, color fundus photography, ocular ultrasound, optical coherence tomography (OCT), fluorescein angiography (FFA), and indocyanine green angiography (ICGA). The following demographic data were collected: age at onset, sex, diagnosis, date of known systemic cancer diagnosis, ocular symptoms at presentation, duration, date of first visit, total follow-up duration, and the presence of extraocular systemic metastases before or after the diagnosis of CM. Furthermore, treatment modalities, treatment responses, complications, and the development of new ocular metastases during follow-up were documented for each patient.

### 2.2 Treatment

Ophthalmologic treatment guidelines: Treatment decisions were based on a comprehensive evaluation of the patient’s overall health, cancer prognosis, visual symptoms, location and extent of intraocular lesions, and patient preferences. (1) For patients with stable systemic conditions, asymptomatic ocular involvement, and flat lesions, regular monitoring was recommended. (2) For patients with progressive systemic cancer and active CMs—particularly those with subretinal fluid and secondary retinal detachment—systemic treatment should be initiated. (3) If visual decline persists during systemic therapy or if subretinal fluid progressively expands, combination local therapy should be considered. (4) In cases of advanced systemic cancer or resistance to systemic therapy, local therapy can be used to alleviate ocular symptoms (see Figure 1 for details).

The potential risks and benefits of various ophthalmic treatments were discussed with the patients, and informed consent was obtained before treatments were administered. The treatment parameters were as follows. (1) Transpupillary thermotherapy (TTT): An 810-nm laser was used, with the spot diameter selected, to ensure full coverage of the lesion. The energy parameters ranged from 150 to 1,000 mW. (2) Radioactive patch therapy (RPT): This method involves localized radiation therapy for intraocular tumors. Accurate placement of the applicator is critical. Under local anesthesia, the applicator was sutured to the sclera over the tumor, extending at least 2 mm beyond the edge of the tumor. The placement time was determined in accordance with the guidelines established by the Collaborative Ocular Melanoma Study (COMS) (8). (3) Intravitreal Bevacizumab (IVB) injection: Under local anesthesia, 0.08 mL of bevacizumab was injected into the vitreous cavity through the pars plana.

### 2.3 Follow-up evaluation and statistical analysis

All patients were followed for at least 3 months. During the follow-up visits, patients underwent VA assessment, indirect ophthalmoscopy, color fundus photography, ocular ultrasound, and OCT. The tumor response to treatment was categorized as follows: (1) Growth was defined as an increase in tumor size compared to the initial diagnosis at follow-up. (2) Stability was defined as no change in tumor size compared to the current size during follow-up. (3) Regression was defined as a reduction in tumor size relative to the current size at follow-up. Recurrence was identified if the initial tumor regressed and then regrew at the same site. A new metastasis was classified as the appearance of a lesion at a site distinct from the primary tumor, as detected by fundus examination. Visual acuity changes were categorized as improved, stable, or deteriorated, with improvement defined as an increase in VA of more than two lines on the Snellen chart and deterioration defined as a decrease of more than two lines.

The data were organized via Microsoft Excel (version 2019; Microsoft Corporation, Redmond, WA, USA). The percentage distribution by sex and primary tumor type was calculated, and central tendency measures (mean, range) for quantitative parameters were determined. The correlation of continuous variables was analyzed via the Mann–Whitney *U*-test, with statistical analysis performed via IBM SPSS Statistics (version 26; IBM Corp., Armonk, NY, USA).

## 3 Results

### 3.1 General condition of the patients

This case series included 10 male and 22 female patients, with ages ranging from 32 to 71 years (mean age: 52.53 ± 10.81 years), reflecting a typical age distribution for CM patients. At presentation, 26 patients had unilateral involvement, while 6 patients had bilateral involvement. A total of 26 patients had been previously diagnosed with systemic cancer, including 11 patients with lung cancer, 13 patients with breast cancer, and 2 patients with esophageal cancer. Six patients initially presented with ocular symptoms and were later diagnosed with lung cancer. All patients presented with ocular symptoms, including decreased vision, visual field obstruction, visual distortion, flashes of light, and eye pain. The initial visual acuity assessment revealed two eyes with no light perception, one eye with light perception, two eyes with hand motion to count fingers vision, four eyes with vision <0.1, five eyes with vision between 0.1 and 0.4, and five eyes with vision between 0.4 and 0.6 (see Table 1 for details).

**Table 1:** General condition of patients with choroidal metastases.

Patient	Sex	age	Eye laterality	Primary Tumor	Time for ophthalmologic consultation	Initial Sx	VA		Initial NCT (mmHg)	Ocular/system Treatment	Metastatic sites (except eye)
							Initial	2 months after treatment			
1	F	66	OU	BC	9 years after the diagnosis of primary tumor	Decreased VA	OD 0.2 OS 0.3	OD 0.2 OS 0.2	OD 13.4 OS 12	IVB, chemotherapy	No
2	M	35	OD	NSCLC	1 year after the diagnosis of primary tumor	Decreased VA	0.15	0.15	12	Chemotherapy	No
3	F	61	OD	BC	17 years after the diagnosis of primary tumor	Decreased VA, lacrimation	0.5	0.5	15	Chemotherapy	No
4	F	67	OS	NSCLC	3 years after the diagnosis of primary tumor	Decreased VA	0.2	0.5	16.8	TTT, targeted therapy	No
5	F	41	OS	BC	5 years after the diagnosis of primary tumor	Decreased VA	0.25	0.01	13	PRT, chemotherapy	Bone, lung
6	F	37	OS	NSCLC	Diagnosed at ophthalmology first	Decreased VA, visual field defect	0.01	0.01	12	PRT, chemotherapy	No
7	F	53	OS	NSCLC	1 year after the diagnosis of primary tumor	Decreased VA, visual field defect, Ocular pain	0.3	0.3	8.8	IVB, targeted therapy	IM
8	M	53	OS	EC	3 years after the diagnosis of primary tumor	Decreased VA	0.3	0.3	10	PRT, chemotherapy	Lung, liver
9	F	41	OU	NSCLC	Diagnosed at ophthalmology first	Decreased VA, aethomma	OD 1.0 OS 0.15	OD 1.0 OS 0.15^+1^	OD 16 OS 16	PRT	No
10	F	56	OU	BC	18 years after the diagnosis of primary tumor	Decreased VA	OD 0.15 OS 0.15	OD 0.15 OS 0.15	OD 17 OS 16	Chemotherapy	Lung, adrenal gland, lymph node, bone
11	M	39	OS	NSCLC	1 year after the diagnosis of primary tumor	Decreased VA	0.3	0.16	15	IVB	No
12	F	39	OU	BC	6 years after the diagnosis of primary tumor	Decreased VA, visual field defect, metamorphopsia	OD NLP OS NLP	OD NLP OS HM/30 cm	OD 13 OS 10	PRT	Brain
13	M	71	OD	EC	3 years after the diagnosis of primary tumor	Decreased VA, visual field defect	CF/20 cm	0.03	20	PRT	Lung
14	F	61	OD	NSCLC	2 years after the diagnosis of primary tumor	Decreased VA	0.05	0.1	10.5	IVB	No
15	F	47	OD	BC	4 years after the diagnosis of primary tumor	Decreased VA	0.01	HM	11	Chemotherapy	Bone, lymph node
16	F	58	OD	BC	8 years after the diagnosis of primary tumor	Decreased VA	0.5	0.6	19	TTT	No
17	F	52	OD	BC	8 years after the diagnosis of primary tumor	Decreased VA	NLP	NLP	19	IVB	Lung
18	F	32	OS	BC	6 years after the diagnosis of primary tumor	Decreased VA, visual field defect, metamorphopsia, ocular pain	CF/10 cm	0.3	8.6	IVB, targeted therapy	Bone, bone, liver
19	M	47	OD	NSCLC	Diagnosed at ophthalmology first	Decreased VA, metamorphopsia, ocular pain	0.5	0.6	16.1	TTT	No
20	F	51	OS	NSCLC	Diagnosed at ophthalmology first	Decreased VA	LP	0.12	17	PRT	No
21	M	49	OS	NSCLC	1 month after the diagnosis of primary tumor	Decreased VA	NLP	HM/10 cm	9	PRT, chemotherapy	Liver, bone, brain, adrenal gland
22	F	46	OD	BC	2 years after the diagnosis of primary tumor	Decreased VA, metamorphopsia	0.2	0.25	14	PRT	Lung, bone, adrenal gland
23	F	55	OD	NSCLC	Diagnosed at ophthalmology first	Decreased VA, visual field defect	0.6	1	12.6	TTT, targeted therapy	Bone, brain, adrenal gland
24	M	67	OD	NSCLC	7 months after the diagnosis of primary tumor	Decreased VA, metamorphopsia	CF/1 m	0.03	15	PRT, chemotherapy	No
25	M	57	OS	SCLC	1 year after the diagnosis of primary tumor	Decreased VA, visual field defect, ocular pain	0.5	0.12	10	PRT	Bone
26	F	64	OD	NSCLC	1 year after the diagnosis of primary tumor	Decreased VA, aethomma, ocular pain	0.4	NLP	13	PRT, chemotherapy	Pleura
27	F	70	OU	BC	7 years after the diagnosis of primary tumor	Decreased VA	OD 0.1 OS 0.1	OD 0.1 OS 0.1	OD 7 OS 7	PRT	Lung
28	F	67	OD	NSCLC	2 years after the diagnosis of primary tumor	Decreased VA	OD 0.1	0.05	19	PRT	No
29	M	58	OD	NSCLC	Diagnosed at ophthalmology first	Decreased VA, visual field defect	1.0	1.0	15	Targeted therapy	Bone, IM
30	M	61	OD	NSCLC	4 month after the diagnosis of primary tumor	Decreased VA, visual field defect, ocular pain	0.5	0.25	8	PRT	No
31	F	46	OU	BC	5 years after the diagnosis of primary tumor	Decreased VA, visual field defect, aethomma, metamorphopsia	OD LP OS LP	OD LP OS HM/10 cm	OD 13 OS 10	PRT	Bone, lung
32	F	58	OS	BC	12 years after the diagnosis of primary tumor	Decreased VA, visual field defect	0.02	CF/15 cm	9	IVB	No

Sx, symptom; F, female; M, male; OU, Oculus Uterque, both eyes; OD, Oculus Dexter, right eye; OS, Oculus Sinister, left eye; VA, visual acuity; LP, cannot detect light; HM, able to detect hand motion; CF, able to count fingers; NSCLC, non-small cell lung cancer; SCLC, small cell lung cancer; BC, breast cancer; EC, esophageal cancer; IM, Intrapulmonary metastasis.

### 3.2 Findings from indirect ophthalmoscopy and fundus photography

**Figure 2:**
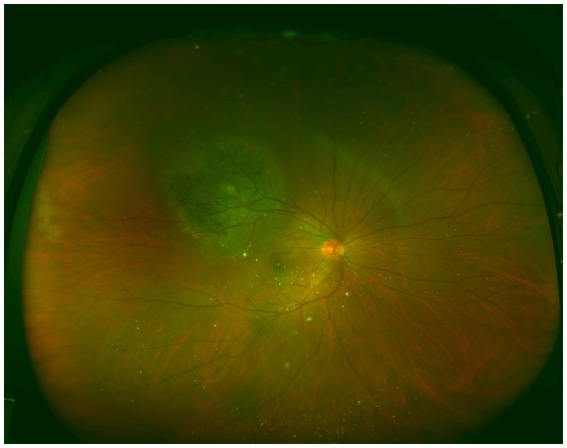
Ultra-widefield color fundus image of an eye affected by choroidal metastases. A round elevated lesion is observed in the superotemporal region, with distinct pigmentation present on the surface of the tumor.

Metastatic cancer was located in the ciliary body and choroid in one eye, whereas in the remaining 37 eyes, the lesions were confined to the choroid. CMs presented as subretinal, pale yellow, or gray-yellow flat elevations with a wide base. The surfaces of the lesions appeared mostly flat but sometimes undulated, with varying degrees of elevation and indistinct borders. Subretinal fluid was observed in some cases, and brown pigmentation was noted on the surface of certain tumors (Figure 2). Lesions associated with primary lung cancer primarily appeared pale yellow, whereas those linked to breast cancer were predominantly milky white. At the initial visit, 36 eyes (94.74%) had a single lesion, while 2 eyes (5.26%) had multiple lesions. All of these lesions were detected among breast cancer patients.

### 3.3 Imaging characteristics

Detailed analysis of ocular ultrasound, OCT, FFA, and ICGA revealed the distinctive features of CM that are crucial for accurate diagnosis and treatment planning.

#### 3.3.1 Ocular ultrasound

The primary features included flat, wide-based lesions or irregular, undulating surfaces, with a few cases displaying round-shaped growth. Tumor necrosis was frequently observed within the lesions, as indicated by heterogeneous internal echoes of varying intensity, with some areas presenting as echo-free dark zones. Exudative retinal detachment was present in 81.58% of the affected eyes, with 28 eyes showing localized retinal detachment surrounding the metastatic lesions and 3 eyes exhibiting extensive retinal detachment. Flat or irregularly undulating lesions were identified in 35 eyes (92.11%), whereas round-shaped lesions protruding into the vitreous cavity were noted in 3 eyes (7.89%). At the initial diagnosis, ultrasound measurements indicated that the height of the CMs ranged from 1.21 to 9.8 mm, with an average of 3.02 mm. The base diameter of the lesions, which can be measured in the affected eyes, ranged from 5.00 to 18.48 mm, with an average of 11.09 mm.

#### 3.3.2 OCT examination

**Figure 3:**
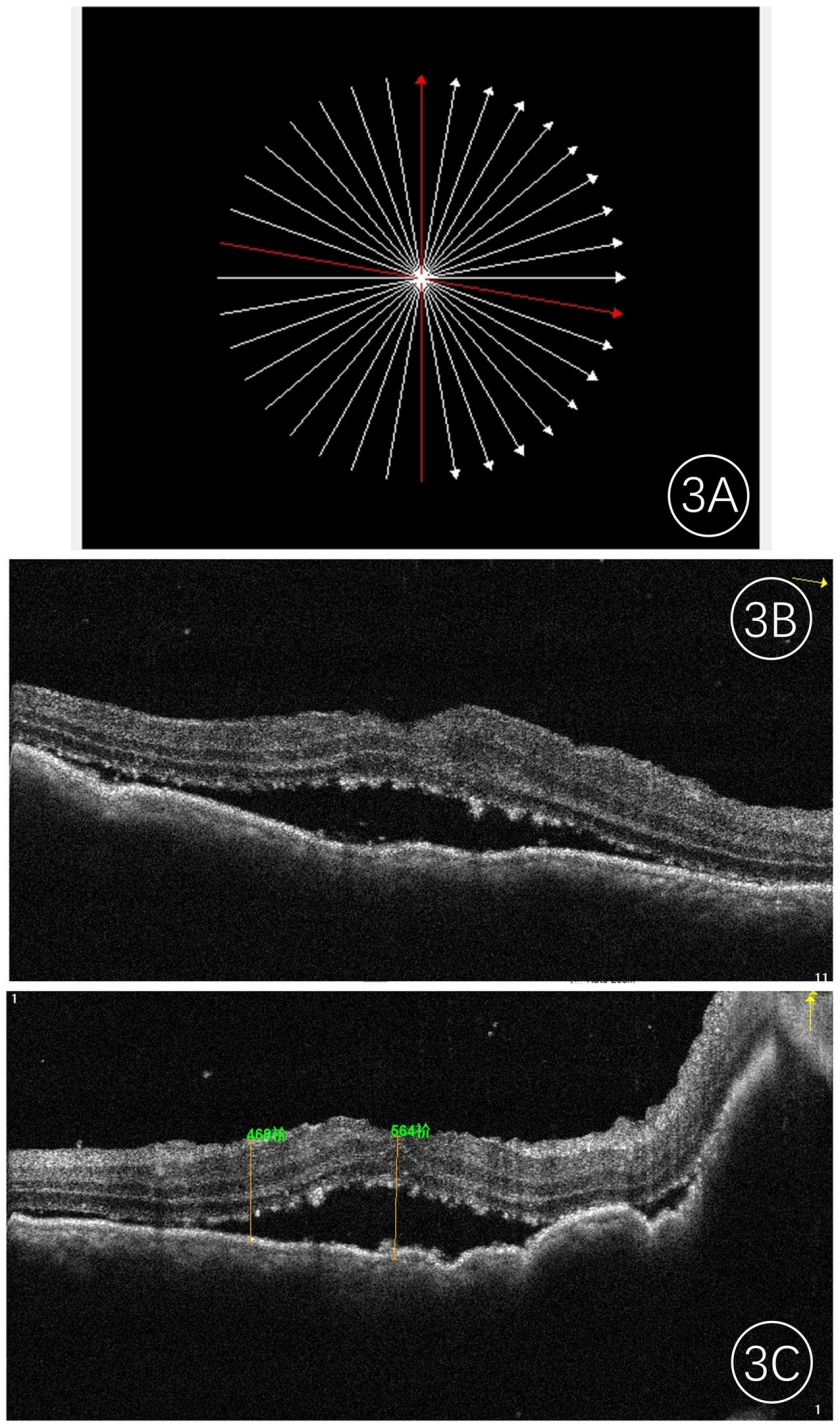
Frequency-domain OCT images of an eye with choroidal metastases. **(A)** Shows the scan location; **(B,C)** present the corresponding OCT images along the red line, revealing a hyporeflective mass with a typically irregular anterior surface, accompanied by retinal pigment epithelium (RPE) changes and speckled shallow subretinal fluid. The central foveal thickness (CFT) was measured at 564 μm.

The most prominent feature observed on OCT was the irregular or undulating anterior surface of the lesion, along with the thickening of the retinal pigment epithelium (RPE) layer. The RPE exhibited varying degrees of wavy changes, and the choroid displayed localized elevation with obscured vascular structures. Localized edema and detachment of the neurosensory retina were noted, with numerous fine granular and clumped hyperreflective deposits visible within the outer retinal layers (Figure 3). In cases of exudative retinal detachment, low-reflective signals were observed beneath the neurosensory retina.

#### 3.3.3 FFA/IGGA examination

**Figure 4:**
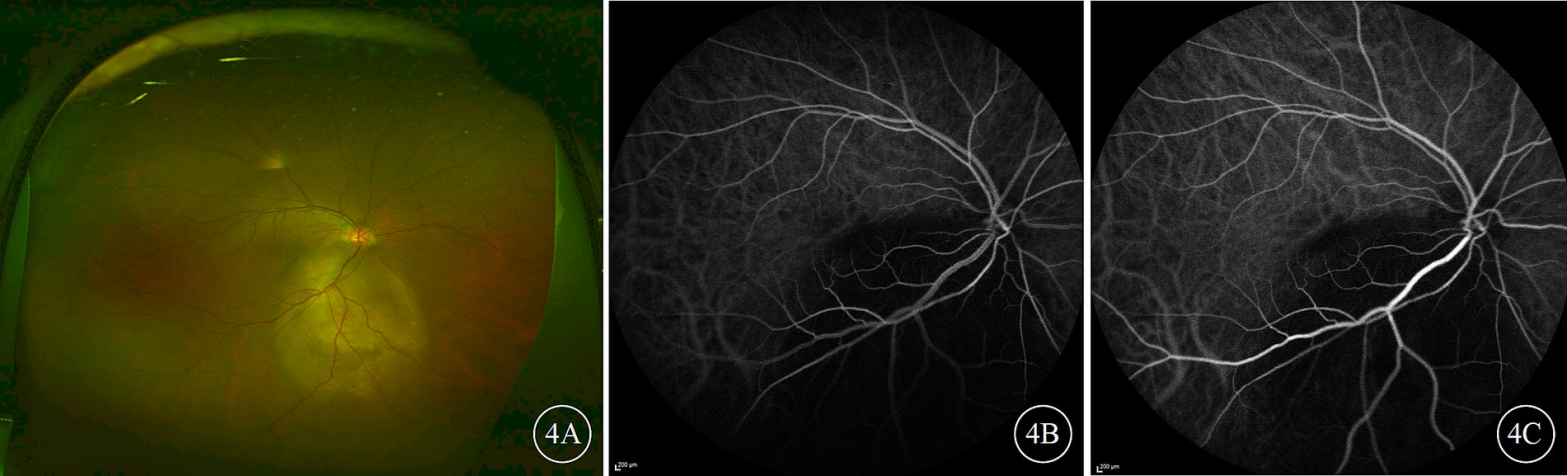
Ultra-widefield color fundus and indocyanine green angiography (ICGA) images of the right eye of a patient with choroidal metastases from lung cancer. **(A)** Presents the color fundus image; **(B,C)** depict early and late-phase ICGA images, respectively, both of which show the tumor lesion as being hypofluorescent.

The FFA results indicated that the filling times of the retinal arteries and veins were generally normal. In the early phase, the choroidal tumor blocked the background fluorescence, and from the arterial to arteriovenous phase, punctate, pinpoint, and patchy hyperfluorescence appeared at the tumor margins, gradually intensifying and merging. In the late phase, the lesion area exhibited mottled hyperfluorescence, and fluorescence leakage was observed over time. IGGA revealed examination showed that the tumor lesions exhibited hypofluorescence and blocked fluorescence in both the early and late phases (Figure 4), with some cases showing a small amount of mottled hyperfluorescence in the late phase. The blood vessels within the tumor exhibited mild staining and leakage; if the tumor was flat and thin, the underlying choroidal vessels were visible through the tumor, clearly delineating the tumor’s extent and abnormal choroidal vessels.

### 3.4 Treatment methods and follow-up results

In this study, the follow-up period ranged from 3 months to 4 years, with a median duration of 4 months. Five patients (six affected eyes) in the study group had small lesions that did not involve the visual function area and were treated with systemic chemotherapy or targeted therapy with regular follow-up. At the 3-month follow-up, an ultrasound examination of the six eyes treated with systemic therapy alone revealed no significant change in lesion height, suggesting that the treatment was effective in terms of stabilizing the lesions without further progression. In one patient with bilateral involvement, subretinal fluid (SRF) was significantly absorbed in one eye, and the lesion height in the other eye decreased by 21.54% compared to that before treatment.

Patients with significant ocular symptoms or rapidly progressing lesions, as determined by clinical evaluation and imaging findings, were selected for local treatment interventions. Among the 32 eyes, 8 received intravitreal bevacizumab (IVB) injections. Four eyes received 2–3 treatments during the follow-up period. At the 2-month follow-up after the last treatment, one eye remained stable, whereas three eyes presented a significant reduction in tumor thickness, with an average decrease of 14.46% (median 13.26% [range 5–63.03%]). The remaining four eyes received only one IVB injection combined with systemic chemotherapy or targeted therapy. Two months after treatment, the tumor base diameter did not increase further, the SRF resolved, and the average tumor thickness decreased by 27.35% (median 11.33% [range 8.45–63.03%]). Two patients with primary lung cancer later experienced rapid progression of ocular lesions due to deteriorating systemic health, leading to enucleation at 24 months and 37 months after local ocular treatment, respectively.

Eighteen eyes underwent proton beam radiation therapy (PRT), and at the 2–3-month follow-up, the ultrasound results indicated that the lesion size was controlled by these patients. Among them, five eyes that received combined systemic chemotherapy presented an average tumor thickness reduction of 31.26% (median 20% [range 11.11–67.27%]). Among the remaining 13 eyes, 10 exhibited a reduction in tumor thickness, and subretinal fluid was absorbed, with an average reduction in tumor thickness of 36.44% (median 35.26% [range 16.56–60.92%]). Two eyes developed secondary glaucoma 3 and 4 months posttreatment, indicating the need for prompt medical intervention to manage intraocular pressure and prevent further vision loss.

Four eyes underwent two sessions of TTT, resulting in controlled tumor height with no further progression. This finding indicated that TTT can effectively stabilize small choroidal metastases, particularly when it is combined with targeted systemic therapy. Among these, two eyes that received combined targeted therapy showed significant reductions in tumor thickness at the 2-month follow-up, with decreases of 23.08 and 70.67%, respectively. One eye recurred during the follow-up period and was subsequently controlled with IVB injection. Two patients with bilateral involvement had only one eye treated due to the stable condition of the other eye, which was monitored. No cases of radiation retinopathy, infection, or postoperative hemorrhage were observed during the follow-up period.

## 4 Discussion

Advances in medical science have led to increased survival rates for cancer patients, thereby increasing the incidence of CM. While CM is typically associated with systemic cancer spread, it may also represent the initial symptom of an unknown primary tumor or the first sign of recurrence in a previously diagnosed primary cancer. The clinical diagnosis of CM primarily depends on the patient’s medical history, clinical presentation, indirect ophthalmoscopic examination, and multimodal imaging techniques (9). In cases where patients present with ocular symptoms but lack a known tumor history, comprehensive systemic cancer screening, in collaboration with relevant specialties, is essential. When CM and primary choroidal melanoma are difficult to differentiate or when systemic workups fail to identify the primary malignancy, a biopsy can provide cytological evidence to distinguish between metastatic and primary tumors (10). To ensure adequate diagnostic information, a biopsy typically requires the lesion to be more than 3 mm in size (11). Although biopsy is considered the most reliable method for tumor diagnosis, some researchers express concerns about the potential dissemination of tumor cells and local complications. Consequently, ocular biopsy may not be necessary in patients with known primary tumors and multiorgan metastasis.

Primary malignancies of various organs can induce secondary CM. Numerous studies from different countries have reported that breast cancer is the most common primary source of CM, followed by lung cancer (5). In contrast, reports from China have indicated that lung cancer is the most common primary tumor, followed by breast cancer and gastrointestinal tumors (10%); other primary tumors are relatively rare (12). This discrepancy may be attributed to the higher incidence of lung cancer and lower early diagnosis rates in China (13). In the current cohort, all cases were secondary to thoracic tumors, with lung cancer accounting for the highest proportion (53.125%), followed by breast cancer (40.625%) and esophageal cancer (6.25%).

Some studies have suggested that because the early symptoms of lung cancer are mild, many patients are already in the late stages of the disease by the time symptoms prompt them to seek medical attention, with metastasis often being the first indication of lung cancer (14). Unlike other primary tumors, lung cancer metastasis is usually detected before the symptoms or diagnosis of the primary tumor (15, 16). In our cohort, all six patients who initially presented to ophthalmologists were later diagnosed with lung cancer, suggesting that among patients with CM and no known history of cancer, lung cancer is the most likely primary tumor. Consequently, if a patient with suspected CM has no prior cancer diagnosis, comprehensive lung cancer screening should be conducted.

The average interval between the diagnosis of the primary lung tumor and CM is shorter than that for breast cancer (6, 17). In our cohort, the mean duration from systemic cancer diagnosis to ophthalmology consultation for the 11 lung cancer patients was 1.2 ± 0.89 years, whereas, for the 13 breast cancer patients, the mean duration was (8.23 ± 4.8) years. Two esophageal cancer patients sought ophthalmological consultation within 3 years of their cancer diagnosis.

Previous studies have indicated that 55–70% of patients with CM experience blurred vision (10, 18). In this study, all patients presented with varying degrees of visual impairment upon initial consultation. This impairment could be attributed to the involvement of the macula or the retinal region surrounding the optic disk or to the occurrence of exudative retinal detachment. Clinical and epidemiological features appear to be useful for determining the type of primary tumor. Meziani et al. reported that 67% of choroidal metastasis cases originating from lung cancer are typically unilateral and involve a single lesion (19), whereas bilateral, multifocal, and diffuse forms are more commonly associated with breast cancer (10, 17, 20). In this case series, 35 out of 38 eyes had solitary lesions, 3 had multiple lesions, and 6 patients had bilateral involvement. Notably, all cases of multifocal and bilateral involvement were associated with primary breast cancer.

The fundus characteristics of CM include certain clinical features, typically presenting as flat, elevated lesions that appear gray-yellow or milky-white. In some cases, fundus examination may be hindered by refractive media opacity or retinal detachment. However, ocular ultrasound, which is not affected by media opacity, remains a crucial diagnostic tool in clinical practice. The findings of this study are consistent with those of previous studies, with the majority of affected eyes showing flat or irregularly wavy intraocular masses. A few lesions displayed round growth, often accompanied by localized or extensive retinal detachment. In rare instances, the metastasis may cause a rupture of Bruch’s membrane, resulting in a mushroom-shaped lesion (21, 22). Previous studies have reported that the mean height of tumors in 724 CM-affected eyes was 3.4 mm, with an average horizontal base diameter of 9.7 mm (23). In the present study, the initial ultrasound measurements revealed an average thickness of 3.02 mm, with a measurable basal horizontal diameter averaging 11.09 mm.

OCT is highly effective in visualizing the morphology, size, and condition of the surrounding retinal structures of a tumor, making this imaging approach an indispensable tool in both the initial diagnosis and ongoing monitoring of choroidal metastases. The sensitivity of OCT surpasses that of other diagnostic devices, especially in terms of detecting small tumors in the posterior pole and subtle changes in the choroid (24, 25). In this patient cohort, OCT images revealed surface undulations of the tumor, RPE thickening, and varying degrees of wave-like elevations in both the choroid and RPE layers. Additionally, fine granular or clustered hyperreflective material was observed between the neuroepithelial layer and the RPE, whereas exudative retinal detachments were characterized by hyporeflective signals beneath the neuroepithelial layer. These findings were consistent with those reported by Vishnevskia-Dai et al. (26). In patients who responded to treatment, follow-up OCT revealed a gradual decrease in tumor thickness, with some lesions flattening and disappearing, along with an increase in internal reflectivity.

FFA is not a conclusive method for diagnosing CM, as most choroidal tumors display similar features on angiography (26). In contrast, ICGA has significant value for the differential diagnosis of CMs. These metastases typically appear as hypofluorescent lesions at all stages and can reveal a larger area of choroidal involvement than FFA (3, 27). Consequently, while FFA is useful for evaluating changes in the RPE, ICGA provides a more detailed assessment of tumor extent and abnormalities in the choroidal vasculature.

The presence of CM in tumors is indicative of advanced disease. The primary objectives of treatment are to preserve or improve vision, prevent enucleation, maintain quality of life, and prolong survival. Systemic therapies, including chemotherapy and targeted treatments aimed at the primary tumor, have also been shown to exert therapeutic effects on ocular metastases. A study examining 12 patients (1995–2014) who received only systemic chemotherapy without local treatment reported that at least two patients had isolated CM without additional distant metastasis. Among these patients, eight (66.7%) demonstrated regression of CM following treatment (28). Another study involving 22 lung cancer patients treated exclusively with chemotherapy reported that 68% experienced CM regression, whereas only 20% of 15 eyes treated with observation alone experienced lesion regression (6). In the present study, five patients who underwent only systemic therapy demonstrated stable lesions during follow-up, with no recurrence observed at the final visit.

Although systemic therapies can partially control tumor progression (29), local treatments are particularly critical for patients experiencing vision loss due to tumors or for those unresponsive to systemic therapies (30). Ocular local therapies include laser treatments (TTT, PDT), radiation therapies (PRT, proton beam therapy, stereotactic radiation), and intravitreal injections of anti-vascular endothelial growth factor (VEGF) agents (20, 31). In our study, laser therapy was considered for isolated lesions with a basal diameter of ≤10 mm and a thickness of ≤4 mm (32). Radiation therapy is primarily used for larger or multifocal lesions that are difficult to manage with laser treatment. IVB injections are considered for patients with neovascularization, retinal edema, or those with poor general health who cannot tolerate more complex treatment regimens.

For patients with a single small lesion, TTT offers a rapid and relatively cost-effective treatment option, significantly reducing the time spent in a medical setting. In this study, four eyes treated with TTT had single, unilateral lesions, each smaller than 3.5 mm, with minimal SRF, and the lesions remained stable after treatment. A commonly used local radiation treatment for ocular tumors involves the application of a scleral plaque, where a radioactive particle source is surgically implanted on the external surface of the sclera for localized radiation therapy. Shah et al. (6) examined a cohort of 19 lung cancer patients with CMs treated with PRT and found that 18 patients experienced varying degrees of improvement. In this study, plaque radiotherapy successfully controlled the tumor size in all 18 treated eyes, with the majority of patients showing improvements in vision. The hospitalization period for plaque radiotherapy did not exceed 1 week. The majority of the literature indicates that, following the first IVB injection, tumors in treated eyes show partial regression within a few weeks or complete regression after prolonged follow-up and additional injections (33). In the present study, at 2–3 months post-IVB injection, SRF was reduced in some patients, the tumor volume was controlled at 12.5, and 87.5% of tumors exhibited regression. Eyes treated with combined chemotherapy exhibited significantly greater improvements (*p* < 0.05).

In conclusion, due to the increasing incidence of CM, early diagnosis and management of the condition are essential. For CMs that exhibit favorable responses to systemic treatment, periodic monitoring without ocular intervention may be appropriate. However, if the metastases increase in size during systemic therapy or are the sole site of distant metastasis, local ocular treatment should be considered. The treatment strategy for CM requires interdisciplinary collaboration. The selection of an appropriate treatment plan should take into account the patient’s overall health, the location and number of primary tumors, the extent of ocular involvement, and the presence and activity of other distant metastases, all of which are critical to improving the patient’s quality of life. However, our study has certain limitations. The follow-up period was relatively short, and only short-term efficacy was observed without assessing the long-term effects of treatment or potential recurrence risks. Future research should further extend the follow-up period to comprehensively evaluate the long-term effects and recurrence rates. Additionally, future studies should optimize diagnostic algorithms to differentiate primary from metastatic tumors earlier and refine systemic and local treatment regimens to improve patient outcomes and quality of life.

## Data Availability

The original contributions presented in the study are included in the article/supplementary material, further inquiries can be directed to the corresponding author.
